# Maternal and perinatal mortality: geospatial analysis of inequality in pregnancy related and perinatal mortality in Ethiopia

**DOI:** 10.1093/heapol/czae122

**Published:** 2024-12-17

**Authors:** Sisay Mulugeta Alemu, Gerd Weitkamp, Abera Kenay Tura, Kerry LM Wong, Jelle Stekelenburg, Regien Biesma

**Affiliations:** Global Health Unit, Department of Health Sciences, University Medical Center Groningen, Groningen, Groningen 9713 GZ, The Netherlands; Department of Cultural Geography, Faculty of Spatial Sciences, University of Groningen, Groningen, Groningen 9747 AD, The Netherlands; School of Nursing and Midwifery, College of Health and Medical Sciences, Haramaya University, Haramaya, Ethiopia; Department of Obstetrics and Gynaecology, University Medical Centre Groningen, University of Groningen, Groningen, Groningen 9713 GZ, The Netherlands; Department of Infectious Disease Epidemiology, Faculty of Epidemiology & Population Health, London School of Hygiene and Tropical Medicine, London, London WC1H 9SH, United Kingdom; Global Health Unit, Department of Health Sciences, University Medical Center Groningen, Groningen, Groningen 9713 GZ, The Netherlands; Department Obstetrics & Gynaecology, Leeuwarden Medical Centre, Leeuwarden, Leeuwarden 8934 AD, The Netherlands; Global Health Unit, Department of Health Sciences, University Medical Center Groningen, Groningen, Groningen 9713 GZ, The Netherlands

**Keywords:** pregnancy-related mortality, perinatal mortality, geospatial analysis, Ethiopia, sub-Saharan Africa

## Abstract

While there is ample evidence of the overall reduction in perinatal and pregnancy-related mortality in Ethiopia, it remains uncertain if geographic disparities have diminished. This study aimed to investigate perinatal and pregnancy-related mortality spatial distributions, trends over time, and factors associated with the distribution in Ethiopia. We used data from Ethiopian Demographic and Health Surveys conducted in 2000, 2005, 2011, and 2016. In each survey, around 15 500 women aged 15–49 years were interviewed from about 550 neighborhoods randomly sampled from across the country. Perinatal and pregnancy-related mortality were used as outcome variables. We carried out an optimized hotspot analysis using the Getis-Ord Gi* statistic in ArcGIS Pro to identify the time trend of geographical clusters with high (hot spot) and low (cold spot) perinatal and pregnancy-related mortality. In addition, we conducted a geographically weighted Poisson regression in R to examine the factors associated with the spatial distribution of perinatal and pregnancy-related mortality. Perinatal and pregnancy-related mortality exhibited a clustering pattern, indicating the presence of geographic inequality, with a decreasing pattern from 2000 to 2016. We detected hotspot areas in developed administrative regions of Amhara, Oromia, and Southern Nations, indicating inequality within large regions. Inequality in perinatal mortality was associated with rural residence, younger age of women, and high birth rate, whereas pregnancy-related mortality was associated with low autonomy, younger age, and anemia. We found that anemia (*P*-value = .01) has a geographically varying relationship with perinatal mortality, while education (*P*-value = .03) and wealth (*P*-value = 0.01) are associated with pregnancy-related mortality. While there has been a reduction during the study period, geographical disparities in perinatal and pregnancy-related mortality still persist. Therefore, targeting intervention programs in areas where spatial inequalities still persist is essential for effectively utilizing scarce resources.

Key messages

- Perinatal and pregnancy-related mortality are geographically clustered, signifying geographic inequality in Ethiopia.
- Geographical disparities decreased between 2000 and 2016.
- Central regions like Amhara, Oromia, and Southern Nations Nationalities and People’s Region had higher mortality clusters.
- Rural living, younger age, and higher birth rates influenced perinatal mortality distribution, while lower autonomy, younger age, and anemia correlated with pregnancy-related mortality distribution.

## Introduction

Reducing maternal and perinatal mortality is a high-priority international goal (United Nations 2015). Although there is global progress in reducing maternal and perinatal mortality, the levels are still unacceptably high, especially in low- and middle-income countries (LMICs). Almost all (99.5%) maternal deaths occur in LMICs, with 71.8% occurring in sub-Saharan African countries, including Ethiopia. Ethiopia has made significant progress in reducing maternal and perinatal mortality since the adoption of the Millennium Development Goals (World Health Organization 2019). However, the maternal mortality ratio (267 per 100 000 live births) and perinatal mortality rate (33 per 1000 live births) are still high (Jena et al. 2020, United Nations Maternal Mortality Estimation Inter-agency G 2023) and could be reduced if women and newborns receive timely and quality care (Federal Democratic Republic of Ethiopia Ministry of Health 2016).

Over the past two decades, Ethiopia has made significant strides in improving its healthcare system, with a particular focus on maternal and child health. To address the needs of rural and hard-to-reach communities, the government has implemented three innovative interventions: Health Extension Program, Health Development Army, and Maternity Waiting Homes. The Health Extension Program was launched in 2003, primarily in rural areas, with health posts staffed by two female health extension workers, as a strategy to move toward universal primary healthcare coverage (Wang et al. 2016). The priorities of this program are to identify pregnant mothers, provide antenatal care, connect women with health centers for facility delivery, and ensure the provision of postnatal care. The Health Development Army is a women-centered community organization that works closely with health extension workers to encourage their members to use maternal health services, among other tasks (Wang et al. 2016). Maternity Waiting Homes provide temporary accommodation during the final weeks of pregnancy to women with high-risk pregnancies and/or living in remote areas by offering residence within or close to hospitals or health centers. Once labor starts or complications arise, women can easily access the facility for childbirth (van Lonkhuijzen et al. 2009).

While the impact of these initiatives on reducing maternal and perinatal mortality is well documented (Banteyerga 2011, Wang et al. 2016, Dadi et al. 2018, Rieger et al. 2019), their impact on reducing inequality is unclear. A modeling study attributed 46% of Ethiopia’s overall decline in the maternal mortality ratio between 2003 and 2016 to the combined effect of the Health Extension Program and Health Development Army (Rieger et al. 2019). Studies also indicated that Maternity Waiting Homes have significantly reduced maternal mortality and stillbirths (Dadi et al. 2018). However, the extent to which these initiatives have reduced geographical inequality in Ethiopia is less documented.

Understanding the time trend of inequality is crucial for recognizing whether progress is being made toward reducing disparity. Moreover, identifying geographic areas where inequalities persist is essential for targeting intervention programs in vulnerable communities and underserved populations. Studies indicate that interventions implemented in underserved areas could save up to eight times more maternal and perinatal lives than when implemented in other areas (Ruhago et al. 2012). In addition, by addressing the factors associated with inequality, we can work toward creating a more just and equitable healthcare system. Therefore, this study aims to (I) map inequalities in perinatal and pregnancy-related mortality and trends of these inequalities over time in Ethiopia and (II) explore factors associated with these inequalities. As a result, we formulated the following research questions: (I) what are the spatial patterns of perinatal and pregnancy-related mortality hotspot areas—clusters of areas with high mortality—in Ethiopia, and how have these patterns evolved over time? (II) What are the factors related to the inequality of perinatal and pregnancy-related mortality in Ethiopia?

## Methods

### Population and data source

We used publicly available data from four consecutive Ethiopian Demographic and Health Surveys (EDHS): 2000, 2005, 2011, and 2016. Each survey included a representative sample of 14 072, 14 500, 17 817, and 18 008 households included in 539, 540, 624, and 645 survey clusters (neighborhoods), respectively. All women aged 15–49 years who were either permanent residents of the selected households or visitors who stayed in the household the night before the survey were interviewed. A total of 15 367, 14 070, 16 515, and 15 683 women were interviewed in each subsequent survey, respectively. The detailed sampling procedure of each survey can be found elsewhere (Central Statistical Authority Ethiopia, O.R.C. Macro 2001; Central Statistical Agency Ethiopia, O.R.C. Macro 2006; Central Statistical Agency Ethiopia, I.C.F. International 2012; Central Statistical Agency—CSA/Ethiopia, ICF 2017). In addition, we used secondary data on health facility location and distribution from the Ethiopian Emergency Obstetric and Newborn Care (EmONC) Assessment 2016. This assessment was a census of all private and public facilities across the country, which included 3804 out of a total of 4587 facilities in Ethiopia that met the following criteria: (I) licensed by the government to attend deliveries; (II) attended births in the last 12 months; and (III) were operational, i.e. not closed or under construction. The detailed sampling procedure of the EmONC assessment can be found elsewhere (Ethiopian Public Health Institute 2017). Moreover, population distribution data containing the number of reproductive-age women in every 30 m^2^ area in Ethiopia were obtained from the United Nations Office for the Coordination of Humanitarian Affairs (UNOCHA). The health facility and population distribution data were used to calculate a health facility density score, which was used as a proxy for access to health facilities (Supplementary Fig. S4). Lastly, we used the data on the administrative boundaries of Ethiopia from openAFRICA (openAFRICA. Dec 2021) for spatial analysis and mapping purposes.

### Variables and measurements

#### Outcome variables

Pregnancy-related mortality (i.e. as a proxy for maternal mortality) and perinatal mortality were used as the two outcome variables for this study.

Pregnancy-related mortality, defined by the Demographic and Health Survey (DHS) as the death of a woman while pregnant or within 2 months of termination of pregnancy irrespective of the cause of death (The DHS Program. June 2022), was measured using the sisterhood method (World Health Organization 1997). In the sisterhood method, each woman in selected households was asked about each of her siblings born to the same mother. For deceased female siblings, further information was collected to determine whether the death was pregnancy-related, i.e. whether the sibling died during pregnancy, delivery, or within 2 months after the end of a pregnancy or childbirth. Since all women of reproductive age who stayed in the selected household the night before the survey were eligible to be interviewed, multiple siblings in the same household could be eligible for participation. Thus, the death of a woman could be reported by more than one sibling, leading to double counting of a single death. We removed duplicate cases of pregnancy-related deaths. The algorithm we used for exclusion is available in Supplementary Fig. S1 and Table S1. We then calculated the pregnancy-related mortality ratio as the ratio of total pregnancy-related deaths within 7 years preceding the survey to the total live births in the same period for each neighborhood. This was then used as the outcome variable for the first research question. For the second research question, we take total pregnancy-related deaths in each neighborhood as an outcome variable.

For this study, perinatal mortality refers to having either stillbirth or neonatal death (newborn death within the first month of life), which is different from the World Health Organization definition, the death of a fetus toward the end of a pregnancy or during the first week of life (World Health Organization 2016). This is because we included all newborn deaths up to the first month of life to increase the sample size and, hence, the power of the study. DHS assessed both stillbirths and neonatal deaths through a self-report of the mother. Each woman in selected households was asked about the number of children she has, the date of birth and survival status of each child, age at death for the deceased children, and whether she had a stillbirth. We then calculated the perinatal mortality rate as the ratio of total perinatal death (i.e. total stillbirth plus neonatal death) per 1000 live births within 5 years preceding the survey to the total live births in the same period for each neighborhood. This was then used as the outcome variable for the first research question. For the second research question, we took total perinatal deaths in each neighborhood as an outcome variable.

#### Geographical information

The latitude and longitude coordinates of the geographic center of each neighborhood were collected. For confidentiality purposes, the coordinates of each neighborhood were randomly displaced before public release (Central Statistical Agency—CSA/Ethiopia, ICF. 2017). The maximum displacement was 2 km for urban neighborhoods and 5 km for 99% of rural neighborhoods. The remaining 1% of rural neighborhoods were displaced at a maximum of 10 km (Burgert et al. 2013).

### Data preparation and analysis

Since EDHS collected geographic coordinates at the neighborhood level, the unit of spatial analysis was neighborhoods. We excluded a total of 4 neighborhoods (containing 85 households) from EDHS 2000, 7 neighborhoods (containing 137 households) from EDHS 2005, 25 neighborhoods (containing 487 households) from EDHS 2011, and 21 neighborhoods (containing 365 households) from EDHS 2016 due to missing geographical coordinates. Ultimately, we included 535, 528, 571, and 622 neighborhoods from the 2000, 2005, 2011, and 2016 surveys, respectively. The data were weighted using sampling weights before any statistical analysis to restore the representativeness of the survey. Then, we projected the data from a global geographic coordinate system (WGS84) into a local projected coordinate system, Adindan/Universal Transverse Mercator zone 37N.

We carried out optimized hotspot analysis using the Getis-Ord Gi* statistic in ArcGIS Pro software to identify the presence of statistically significant geographical clusters of neighborhoods that have high values (hot spots) and low values (cold spots) of perinatal and pregnancy-related mortality ratios. The optimization process was applied to determine an optimal search distance that maximizes the identification of statistically significant hotspots. For this study, different distance bands (ranging from 72 to 163 km) were used for each hotspot analysis. The different distance bands used for each analysis can be found in Supplementary Table S2.

In addition, we conducted a Poisson regression followed by a geographically weighted Poisson regression analysis using the most recent data from the four surveys, the EDHS 2016 dataset, to identify the factors associated with the spatial distribution of perinatal and pregnancy-related mortality counts. Our selection of the 2016 dataset aimed to capture the factors relevant to the current or recent distribution of mortality. First, we aggregated variables at the neighborhood level, which was done differently for different variables. For continuous variables (age, years of education, and wealth index) and composite score variables (anemia and circumcision score), the mean neighborhood score was used. On the other hand, the proportion of women in the neighborhood was used for the dichotomous variables (antenatal care, skilled birth attendance, and contraceptive use). The detailed operationalization and categorization of variables can be found in Supplementary Table S3. Then, we used the forward-stepwise method to build a Poisson regression model. We added variables one by one to the model based on the order of importance according to the literature and clinical judgment of the authors in the following order: total birth, skilled birth attendance, antenatal care, service environment score, anemia status, contraceptive use, watching television, internet usage, listening to a radio, age at first birth, age at marriage, circumcision, domestic violence, autonomy, polygamy, age, education level, marital status, wealth index, and residence. Variables were kept in the model if they improved the model performance significantly (Akaike information criterion of two or more), except for sociodemographic variables (age, educational level, marital status, wealth index, and residence), which were kept in the model irrespective of the model performance. Finally, we checked whether the model’s residuals were randomly distributed spatially through Moran’s *I* test. In the case of a significant Moran’s *I* test, which indicates the presence of statistically significant spatial clustering of residuals, we performed geographically weighted Poisson regression in R to check the presence of spatially varying relationships of covariates with perinatal and pregnancy-related mortality. Lastly, we performed the Monte Carlo test of nonstationarity to check whether the geographically varying relationship between covariates and outcome variables is statistically significant.

## Results

### Characteristics of the sample

The number of neighborhoods included varied in each survey. On average, 564 neighborhoods were included in the four subsequent surveys (2000, 2005, 2011, and 2016). Around three quarters of neighborhoods were rural; on average, around 10 500 households were included in each of the four surveys (Table 1).

**Table 1.** Characteristics of neighborhoods and households included in the study

Survey year		2000	2005	2011	2016	Total
Total neighborhoods included		535	528	571	622	2256
Neighborhoods per residence	Rural	397 (74.2%)	384 (72.7%)	408 (71.5%)	420 (67.5%)	1609 (71.4%)
	Urban	138 (25.8%)	144 (27.3%)	163 (28.5%)	202 (32.5%)	647 (28.6%)
Total households included		10 348	9680	11 242	10 843	42 113

### Perinatal and pregnancy-related mortality hotspot areas and trends over time

The global spatial autocorrelation analysis revealed that the observed spatial distribution of perinatal and pregnancy-related mortality in each of the four surveys was statistically significantly different from a random spatial distribution, which indicates that they were not geographically equally distributed across the country and exhibited a clustering effect (Supplementary Table S4).

**Figure 1.**
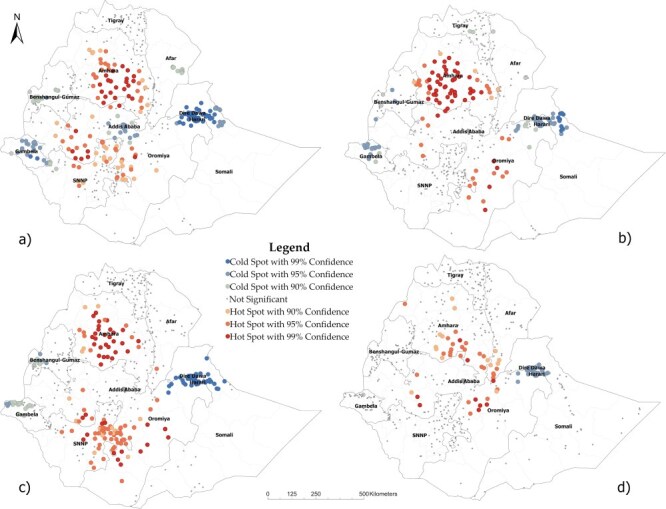
Hotspot and coldspot areas of perinatal mortality in Ethiopia in the first four subsequent DHS: (a) 2000, (b) 2005, (c) 2011, and (d) 2016.

**Figure 2.**
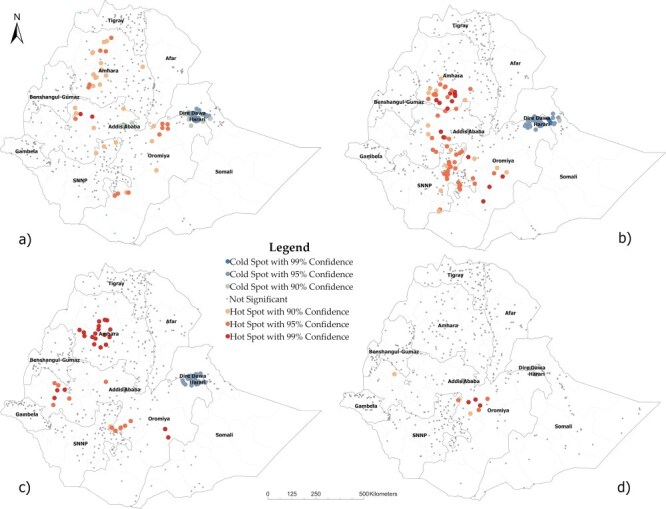
Hotspot and coldspot areas of pregnancy-related mortality in Ethiopia in the first four subsequent DHS: (a) 2000, (b) 2005, (c) 2011, and (d) 2016.

Figures 1 and 2 show the results of hotspot analysis performed to identify areas in Ethiopia with significantly higher and lower perinatal and pregnancy-related mortality, respectively. Each dot on the map represents a neighborhood. The red-colored dots indicate hotspot areas, neighborhoods with a surrounding area with significantly higher mortality than the rest of the country. In contrast, the blue dots indicate coldspots, neighborhoods with a surrounding area with significantly lower mortality than the rest of the country. The brightness of the red and blue colors indicates an increased significance level (i.e. 90%, 95%, and 99% confidence levels). We detected local spatial clusters, i.e. hotspot and coldspot areas, for perinatal and pregnancy-related mortality in each survey.

#### Spatial clusters of perinatal mortality

We found hotspot areas of perinatal mortality in the central and southern Amhara region, central Oromia, the eastern part of the Southern Nations Nationalities and People’s Region, and central Afar in 2000. On the other hand, we found coldspot areas in Addis Ababa, Dire Dawa and Harari, Gambella, and Benishangul-Gumuz. Perinatal mortality hotspot and coldspot locations in 2005 and 2011 were generally similar to what we observed in the 2000 survey. However, the coldspots we observed in Addis Ababa in 2000 disappeared in 2005 and 2011. On the other hand, in 2005, very few clusters of low mortality appeared in the Tigray region but were no longer there in 2011 and 2016. Also, we observed an increase in the number of hotspots in the Southern Nations Nationalities and People’s Region in 2011. In 2016, the number of both hotspots and coldspots in the country decreased. As a result, hotspots remained only in central and southern Amhara and central Oromia, while clusters of low mortality remained only in Dire Dawa and Harari. Meanwhile, new hotspots emerged in the southern Afar region in 2016 (Fig. 1).

#### Spatial clusters of pregnancy-related mortality

In 2000, we observed hotspots around central Amhara, central Oromia, and eastern Southern Nation Nationalities and People Region, while coldspots were in Addis Ababa, Dire Dawa, and Harari. Similar patterns persisted in 2005 and 2011, except the coldspots in Addis Ababa disappeared. In 2016, only a few hotspots and no coldspots were detected. Six of the eight hotspot neighborhoods observed during this period were located in central Oromia, specifically in the Arsi zone (Fig. 2).

## Factors associated with the spatial distribution of perinatal and pregnancy-related mortality

Poisson regression indicated that rural neighborhoods (95% confidence interval, CI = 0.21, 1.31) and a lower average age of women (95% CI = −0.15, −0.04) were found to be associated with higher perinatal mortality. Also, high perinatal mortality was observed in neighborhoods with a high total birth rate (95% CI = 0, 0.01). Moreover, high pregnancy-related deaths were observed in neighborhoods with low women autonomy (95% CI = −1.97, −0.37), a lower average age of women (95% CI = −0.24, −0.04), and higher anemia rates (95% CI = 0.24, 1.4) (Table 2).

**Table 2.** Factors associated with the spatial distribution of perinatal and pregnancy-related mortality in Ethiopia, using DHS 2016 data

	Perinatal mortality		Pregnancy-related mortality	
	Estimate (95% CI)	*P*-value	Estimate (95% CI)	*P*-value
Intercept	0.87 (−0.89, 2.63)	.331	2.7 (−0.42, 5.81)	.089
Age of women	**−0.09 (−0.15, −0.04)**	**.001**	**−0.14 (−0.24, −0.04)**	**.007**
Education status	−0.07 (−0.16, 0.02)	.117	−0.1 (−0.24, 0.05)	.201
Marital status (married/living together)	0.56 (−0.3, 1.42)	.203	−0.42 (−1.89, 1.07)	.579
Wealth index	−0.05 (−0.21, 0.1)	.5	0.14 (−0.14, 0.42)	.32
Residence (rural)	**0.75 (0.21, 1.31)**	**.007**	0.54 (−0.24, 1.33)	.175
Birth history	**0.01 (0, 0.01)**	**.003**	0.01 (0, 0.01)	.209
Skilled birth	0.07 (−0.44, 0.57)	.776	0.13 (−0.79, 1.03)	.773
Anemia	0.17 (−0.17, 0.5)	.331	**0.83 (0.24, 1.4)**	**.005**
Watching television	0.46 (−0.05, 0.97)	.075	–	–
Using internet	−0.79 (−1.88, 0.23)	.14	–	–
Autonomy	–	–	**−1.16 (−1.97, −0.37)**	**.004**

Bold indicates the variable is associated with the outcome variable. Dashes indicate that the variable is not in the final model for this specific outcome variable.

**Figure 3.**
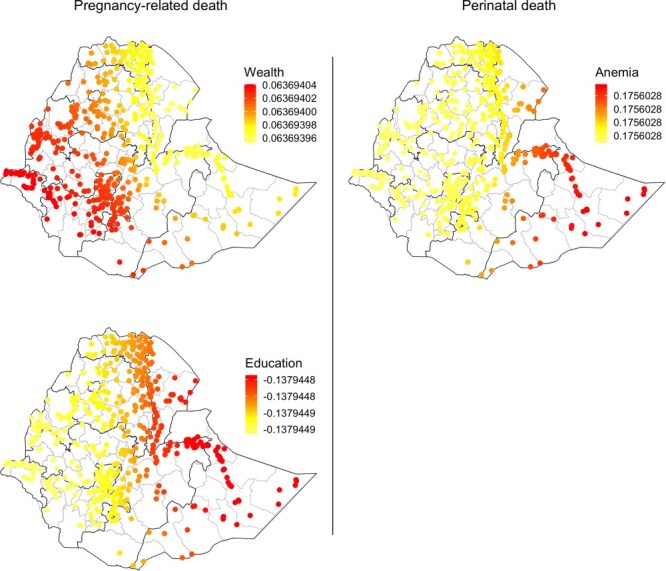
Geographically weighted Poisson regression result of variables that have statistically significant geographically varying relationship with pregnancy-related mortality (left) and perinatal mortality (right) in Ethiopia, using DHS 2016 data (“each dot indicates a neighborhood and the colors represent the regression coefficient locally, i.e. the magnitude and direction of association with the outcome variable at that geographic location”).

Global spatial autocorrelation (Moran’s *I*) showed that the residuals of the perinatal (*P*-value < .001) and pregnancy-related mortality model (*P*-value < .001) were not spatially randomly distributed, suggesting the presence of a geographically varying relationship. Geographically weighted Poisson regression showed that the relationships of perinatal and pregnancy-related mortality with covariates vary geographically, even though these differences are very small. However, the Monte Carlo test of nonstationarity showed that only the geographically varying relationship between a few variables and outcome variables is statistically significant. For example, only anemia’s relationship with perinatal mortality was statistically significant (*P*-value = .01). Similarly, the geographically varying relationship of only education (*P*-value = .03) and wealth (*P*-value = .01) with pregnancy-related mortality was statistically significant (Supplementary Table S5). Figure 3 shows the local regression coefficients of variables that have statistically significant geographically varying relationships with perinatal and pregnancy-related mortality. Mapping these coefficients provides insights into the local magnitude and direction of these associations, as well as their spatial variability across the country. For example, the effect of education on pregnancy-related death is pronounced in the eastern part of the country, while wealth has more effect in the southwestern part of the country. Similarly, anemia has a stronger effect on perinatal death in the eastern part of the country. The local regression coefficient of variables with no statistically significant geographically varying relationship with perinatal and pregnancy-related mortality is shown in Supplementary Figs S2 and S3.

The geographically weighted Poisson regression model for perinatal mortality explains only 16.3% of the variability. Similarly, the pregnancy-related mortality model explains only 8.8% of the variability.

## Discussion

In this study, we first identified perinatal and pregnancy-related mortality hotspot areas and trends over time. Then, we examined factors influencing their spatial distribution in Ethiopia. Both perinatal and pregnancy-related mortality exhibit a spatial clustering pattern, indicating geographical disparities. Higher mortality clusters were found in the central regions of Amhara, Oromia, and the Southern Nations Nationalities and People’s Region. However, we observed a decreasing trend in these disparities from 2000 to 2016. Several factors were found to be associated with the spatial distribution of perinatal mortality, including residing in rural areas, younger age of women, and higher birth rates. On the other hand, factors linked to pregnancy-related mortality included lower autonomy, younger age, and anemia.

The presence of clustering in perinatal and pregnancy-related mortality distribution in Ethiopia indicates geographic inequality. This finding aligns with other analyses using EDHS 2016 data on various maternal and child health outcomes, such as neonatal mortality (Gedamnesh Bitew et al. 2022), stillbirth (Tesema et al. 2020), perinatal mortality (Yadeta et al. 2020), antenatal care utilization (Yeneneh et al. 2018), and postnatal care (Sisay et al. 2019), which also found clustering effects. This confirms the presence of geographic inequality and emphasizes the importance of prioritizing areas with high mortality for resource allocation and targeted interventions.

Between 2000 and 2016, there was a decline in both perinatal and pregnancy-related mortality clusters in Ethiopia, suggesting a reduction in geographic inequality. By 2016, pregnancy-related mortality was distributed relatively evenly throughout the country, with the exception of a few areas in the central Oromia region. Furthermore, recent surveys indicate a decline in urban–rural disparities in neonatal mortality (Shibre et al. 2020). Furthermore, consistent findings from various studies have highlighted a significant reduction in perinatal and pregnancy-related mortality rates in Ethiopia over the past three decades (Ambel et al. 2015, Central Statistical Agency—CSA/Ethiopia, ICF. 2017; Tessema et al. 2017). Ethiopia’s healthcare system has expanded significantly over the past two decades, leading to an increase in the number of health centers and hospitals (Federal Democratic Republic of Ethiopia Ministry of Health 2015). In addition, interventions targeting rural and hard-to-reach communities, such as the Health Extension Program, Health Development Army, and Maternity Waiting Homes, may have contributed to reducing inequality (van Lonkhuijzen et al. 2009, Wang et al. 2016). While the decrease in mortality rates and geographic inequalities reflects the positive trajectory of Ethiopia’s health system, high mortality rates and inequality still persist, requiring further attention. Therefore, it is essential to target these intervention programs in geographic areas where inequalities persist. In addition, it might be important to evaluate the effectiveness of interventions such as the Health Extension Program, Health Development Army, and Maternity Waiting Homes in these specific areas.

We observed higher mortality clusters in economically developed regions of Ethiopia, namely, Amhara, Oromia, and the Southern Nations Nationalities and People’s Region, while lower mortality clusters were observed in eastern Ethiopia and Addis Ababa. These findings partially agree with previous studies on neonatal mortality (Gedamnesh Bitew et al. 2022) and stillbirth (Tesema et al. 2020), which identified hotspot and coldspot areas across the country. The high mortality clusters observed in the central regions may be attributed to hidden local disparities within these regions, which are not captured by surveys like the EDHS and EmONC Assessment as they primarily focus on national and regional averages. Our previous review study highlighted the potential for national- and regional-level summary estimates to obscure marked subnational variations (Alemu et al. 2022). Additionally, despite being classified as economically developed, these regions had poor performance in certain maternal and child health indicators, such as the availability of comprehensive EmONC facilities (Ethiopian Public Health Institute 2017). Conversely, the lower mortality observed in areas around Addis Ababa, Dire Dawa, and Harari could be attributed to better healthcare access and the urban nature of these regions.

Our findings revealed that neighborhoods with higher average birth rates had high perinatal mortality, which is expected as the likelihood of death increases with an increasing number of births (Schultz 1978). Additionally, we observed an association between higher perinatal mortality and rural neighborhoods and neighborhoods with younger women, consistent with previous studies that linked rural residence to stillbirth and neonatal mortality (Kibret et al. 2022). While one study supported the association of younger age with perinatal mortality (Gudayu 2023), two other studies found evidence to the contrary (Yadeta et al. 2020, Gedamnesh Bitew et al. 2022). This inconsistency could be due to the different analysis techniques employed by these studies. For example, some of these studies categorized maternal age, while this study did not. Furthermore, we identified high pregnancy-related mortality in neighborhoods with low women autonomy, low average age of women, and high anemia. There is ample evidence demonstrating the close relationship between women’s decision-making autonomy and maternal healthcare utilization in Ethiopia (Tiruneh et al. 2017). Also, a review study found that 6.37% of maternal mortality in Africa is attributed to anemia (Brabin et al. 2001). Notably, our study did not find an association between clinical factors such as skilled birth attendance, antenatal care, or access to health facilities and perinatal and pregnancy-related mortality, potentially due to our focus on the community (neighborhood) level effect, which may mask individual variations within a community.

The observation that the residuals in both perinatal and pregnancy-related mortality models exhibit a nonrandom spatial distribution indicates spatial clustering, where high values cluster near other high values and low values cluster near other low values. This indicates that a nonspatial linear regression cannot fully explain the association between outcome variables and covariates, as this association naturally varies across space. Geographically weighted Poisson regression showed that the relationships differ geographically. This indicates that the effect of a variable on mortality is not uniform across the country.

Both the perinatal and pregnancy-related mortality models had a low *R*^2^, indicating that the model explains only a small percentage of variability in mortality. This low *R*^2^ may have resulted from aggregating variables at the neighborhood level, obscuring individual variability within neighborhoods. Despite this, the low *R*^2^ reveals how much neighborhood-level variability accounts for variability in mortality across the country. It highlights the complexity and heterogeneity of underlying processes, suggesting that other individual or micro-level factors not captured by our model may also be at play. Nonetheless, analyzing data at the neighborhood level helps us understand the spatial distribution and variation in mortality rates across different neighborhoods, capturing broader sociospatial patterns that might be overlooked with individual-level data.

This study has several strengths and limitations. To the best of our knowledge, this was the first study to pinpoint perinatal and pregnancy-related mortality hotspots over time in Ethiopia. The study used survey data, which is more representative than institution-based (such as hospital-based) mortality data. The study used geospatial analysis to identify inequalities at a subregional level. Moreover, unlike most studies on pregnancy-related and perinatal mortality so far, which focused on individual and clinical variables to explain mortality, the study also included community- and culture-related variables such as polygamy and circumcision to explain the spatial distribution of perinatal and pregnancy-related mortality. However, our study also has limitations that should be considered while interpreting the results. First, DHS measured pregnancy-related mortality indirectly through the sisterhood method. As a result, we do not know where the woman who died due to a pregnancy-related cause actually lived, so we assigned her death to the location where her reporting sibling lives. Second, since women are the ones who reported about their children, women who died were not there to report a perinatal death. Therefore, perinatal deaths from a woman who died could not be included. Third, a small number of neighborhoods were excluded from the analysis due to missing geographical coordinates. Also, the neighborhoods missing geographical coordinates were disproportionately higher in the Somali region. These might affect the representativeness of the sample, although the excluded neighborhoods in each survey were only 0.1–4% of the total neighborhoods. Fourth, since all women in sampled households were interviewed, the death of a woman could be reported by more than one sibling, leading to double counting of a single death. DHS included the double counts to counterbalance under-reporting due to situations where all the siblings in a family are dead, leaving no one to report their death. However, we removed duplicate cases from the analysis because the issue of under-reporting was comparatively less relevant since we are not estimating mortality rates. On the other hand, the issue of multiple counts of the same death would lead to misleading results in the hotspot analysis. Fifth, neighborhood locations were displaced for privacy reasons. This prevented us from accurately measuring how far women live from health facilities. However, we used kernel density estimation to estimate facility density as suggested by DHS, which is less affected by the displacement (Warren et al. 2016). Also, since the neighborhood’s location instead of each household is measured, the unit of analysis in this study was the neighborhood, not individual women. Therefore, it does not show within-neighborhood variability. However, this allowed us to better understand the relationship of perinatal and pregnancy-related mortality at the community level with cultural variables such as polygamy. Lastly, although perinatal and pregnancy-related mortality is still very high in Ethiopia, the mortality figures in each neighborhood are low due to the small nature of the neighborhoods, which decreases the predictive power of the analysis.

## Conclusion

Our study revealed a spatial clustering in perinatal and pregnancy-related mortality in Ethiopia. However, we also observed a consistent decrease in geographical inequality in these mortality rates from 2000 to 2016. This positive trend can be attributed to the government’s prioritization of maternal and child health, as well as targeted interventions such as Maternity Waiting Homes, the Health Development Army, and the Health Extension Program, which specifically address the needs of rural, remote, and hard-to-reach communities. Moreover, we identified relatively higher mortality in the three most populated regions. Therefore, targeting intervention programs in the identified high-risk areas is essential for effectively allocating limited resources. Furthermore, our findings indicated that neighborhoods characterized by high birth rates, younger women, and rural neighborhoods had high perinatal mortality. High pregnancy-related deaths were observed in neighborhoods with low women autonomy, young age, and higher rates of anemia. It is important to note that the strength of these associations varied across different regions of the country. These findings highlight the importance of area-specific strategies and interventions tailored to the unique characteristics and needs of different communities. By addressing the underlying factors contributing to perinatal and pregnancy-related mortality in these specific areas, Ethiopia can further reduce geographical inequalities and improve overall maternal and child health outcomes.

## Supplementary Material

czae122_Supp

## Data Availability

The data underlying this study are available in the article and its online supplementary material.

## Reflexivity statement

To ensure a comprehensive representation of diverse viewpoints and contribute to reducing inequities in global health research, we promoted inclusivity within our authorship. With one female author, two authors originate from low-income countries, and the inclusion of a junior researcher, we sought to achieve a balanced representation.
