# Supplementary material for: Maternal and perinatal mortality: geospatial analysis of inequality in pregnancy related and perinatal mortality in Ethiopia
Source: Health Policy Plan. 2024 Dec 17;40(3):358–67. doi: 10.1093/heapol/czae122 (PMC11886834; doi:10.1093/heapol/czae122)
Supplement: czae122_Supp [file czae122_supp.zip › Supplementary files_Second_revised.docx]

**Supplementary files**

**Table S1:** Algorithm used to handle pregnancy-related death double counts.

| Since all women of reproductive age who stayed in the selected household the night before the survey were eligible to be interviewed, multiple siblings in the same household could be eligible for participation. Thus, the death of women could be reported by more than one sibling, leading to double counting of a single death. We removed duplicate cases of pregnancy-related deaths using the following algorithm. |
| --- |
| 1. First, we automatically included all deaths of siblings, where only one woman was interviewed per household. Then, all other cases were passed to the next algorithm. |
| 1. Next, for households where more than one woman responded, we deduced if the participants from a particular house were siblings or not (born to the same mother) using their relationship to the household head. For example, if only two women were interviewed from a household, and one of them was the wife of the household head, and the other was the daughter/parent/parent-in-law of the household head, we deduced that they were not each other's siblings. We used 12 similar combinations to identify as many households as possible where women who were unlikely to be siblings were interviewed. All deaths of siblings in such a household were included. All other cases were passed to the next algorithm. |
| 1. Finally, for the remaining cases, the response of women from the same household to the following five questions was compared: 1) the number of siblings from the same mother, 2) the number of brothers from the same mother, 3) the number of sisters from the same mother, 4) the number of siblings alive, and 5) the number of siblings dead. If the responses of two or more women from the same household to all the above questions match, those participants were considered siblings. As a result, sibling death from only one of those participants was included. On the other hand, if the answer did not match, we considered them as not siblings, and sibling deaths from all of them were included. As a result, we excluded 939, 743, 707, and 630 women from the 2000, 2005, 2011, and 2016 surveys, respectively (Supplementary Figure S1). |

|  | **2000** | **2005** | **2011** | **2016** |
| --- | --- | --- | --- | --- |
| Pregnancy related mortality | 72.110 | 101.479 | 87.331 | 100.645 |
| Perinatal mortality | 100.688 | 157.072 | 163.891 | 102.201 |

**Table S2:** The different distance bands (in kilometer) used in each optimized hotspot analysis.

**Table S3:** Operationalization and categorization of independent variables.

| *Variable* | *Operational definition* | *Aggregation to neighborhood level* |
| --- | --- | --- |
| Age | Age of the women in years | Mean age of women in the neighborhood |
| Residence | Categorized as either urban or rural | Whether the neighborhood is urban or rural |
| Educational level | Number of years of education in single years | Mean year of education in the neighborhood |
| Marital status | Current marital status (i.e., if a woman is married/living together or not) | Proportion of women married/living together in the neighborhood |
| Listening radio | Ranging from no listening (0) to almost daily (3) | Mean listening radio score in the neighborhood |
| Watching television | Ranging from no viewing (0) to almost daily (3) | Mean watching television score in the neighborhood |
| Internet usage | Ranging from no usage (0) to almost daily in the last month (3) | Mean internet usage score in the neighborhood |
| Total birth | Total number of childbirths a woman has had | Mean number of number of childbirths a woman has in the neighborhood |
| Contraceptive use | Whether a woman is currently using any contraceptive method or not | Proportion of women currently using any contraceptive method in the neighborhood |
| Age at marriage | Age of the woman at her first marriage in years | Mean age at first marriage of women in the neighborhood |
| Age at first birth | Age of the woman when she gives birth for the first time in years | Mean age at first birth of women in the neighborhood |
| Anaemia status | Ranging from no anemia (0) to severe anemia (3) | Mean anemia score in the neighborhood |
| Polygamy | The number of other wives a woman's husband has | Mean number of other wives husbands has in the neighborhood |
| Circumcision | A composite score based on factors such as whether a woman and/or her daughters have been circumcised, whether circumcision is mandatory according to religion, and whether the woman believes circumcision should be stopped | Mean circumcision score in the neighborhood |
| Autonomy | A composite score of degree of independence a woman has in making decisions related to her healthcare, household purchases, visits to family or relatives, use of her husband's earnings, and ownership of her house/land | Mean autonomy score in the neighborhood |
| Domestic violence | A composite score based on 14 questions assessing whether a woman has experienced sexual, physical, or emotional abuse | Mean domestic violence score in the neighborhood |
| Antenatal care | Whether a woman received care from a trained health professional, including community health workers, during her last pregnancy | Proportion of women who received antenatal care during her last pregnancy in the neighborhood |
| Skilled birth attendance | Whether a health professional, including community health workers, assisted with the woman's last delivery | Proportion of women who had a skilled birth attendance during her last delivery in the neighborhood |
| Wealth index | A metric that assesses a household's overall standard of living by taking into account the assets they own, such as televisions, house type, and sanitation facilities. Principal Component Analysis is utilized to create five different wealth index categories (i.e., poorest, poorer, middle, richer, and richest). | Mean wealth index in the neighborhood |
| Health facility density score^*^ | We used the health facility density score as a proxy for access to health facilities. Kernel Density Estimation (KDE) was used to create a map surface with the estimated influence of a given health facility over space, as shown in Figure S4. The further away a facility is located from a neighborhood, the less influence it has and the lower the facility density score at the neighborhood location. Once the KDE is created, the total density score of the area within a 5 km radius of each neighborhood divided by the total population living in the same area was taken as a facility density score of that neighborhood to minimize the impact of displacement. | [This variable was computed for the neighborhood level. Therefore, there was no need for aggregation.] |
| ** We could not use distance to a health facility as random displacement of neighborhoods' geographic coordinates results in measurement error as neighborhoods become closer or farther than their actual distance.* | | |

**Table S4:** Global spatial autocorrelation of perinatal and pregnancy-related mortality

|  |  |  |  |  |  |
| --- | --- | --- | --- | --- | --- |
|  |  | **2000** | **2005** | **2011** | **2016** |
| **Perinatal mortality** | Moran's Index | 0.237 | 0.186 | 0.147 | 0.075 |
|  | Expected Index | -0.002 | 0.002 | -0.002 | -0.002 |
|  | Variance | 0.001 | 0.001 | 0.001 | 0.001 |
|  | z-score | 9.229 | 6.835 | 5.642 | 2.954 |
|  | p-value | 0.000 | 0.000 | 0.000 | 0.003 |
| **Pregnancy-related mortality** | Moran's Index | 0.101 | 0.071 | 0.117 | 0.068 |
|  | Expected Index | -0.002 | -0.002 | -0.002 | -0.002 |
|  | Variance | 0.001 | 0.001 | 0.001 | 0.001 |
|  | z-score | 3.958 | 2.665 | 4.995 | 2.740 |
|  | p-value | 0.000 | 0.008 | 0.000 | 0.006 |

**Table S5:** Results of Monte Carlo Test of non-stationarity that assessed the statistical significance of the spatial variability in the geographically weighted regression coefficients, using Demographic and Health Survey 2016 data.

|  | **Monte Carlo test p-value** | |
| --- | --- | --- |
|  | **Perinatal mortality** | **Pregnancy-related mortality** |
| **(Intercept)** | 0.56 | 0.19 |
| **Age of women** | 0.27 | 0.19 |
| **Education status** | 0.27 | **0.03** |
| **Marital status (Married/living together)** | 0.61 | 0.79 |
| **Wealth index** | 0.95 | **0.01** |
| **Residence (Rural)** | 0.62 | 0.07 |
| **Birth history** | 0.44 | 0.53 |
| **Skilled birth** | 0.98 | 0.07 |
| **Anaemia** | **0.01** | 0.52 |
| **Watching television** | - | 0.19 |
| **Using Internet** | - | 0.29 |
| **Autonomy** | 0.13 | - |

Only one

Include sibling deaths of all women in the household in the analysis

Exclude her siblings' deaths from the analysis

Randomly select one woman from those whose responses match

Include her siblings' deaths in the analysis

A woman's response to all of the following questions matches the response of another woman in the same household.

1. Number of siblings from the same mother,
2. Number of brothers from the same mother,
3. Number of sisters from the same mother,
4. Number of siblings alive, and
5. Number of siblings dead

Number of women interviewed per household?

Using their relationship to the household head, is there a probability that there are women of the same household that are each other's siblings?

More than one

Yes

No

Yes

No

Include her siblings' deaths in the analysis

Woman not selected

Woman selected

Include her siblings' deaths in the analysis

**Figure S1:** Flowchart of the algorithm used for removing duplicate cases of pregnancy-related deaths

**
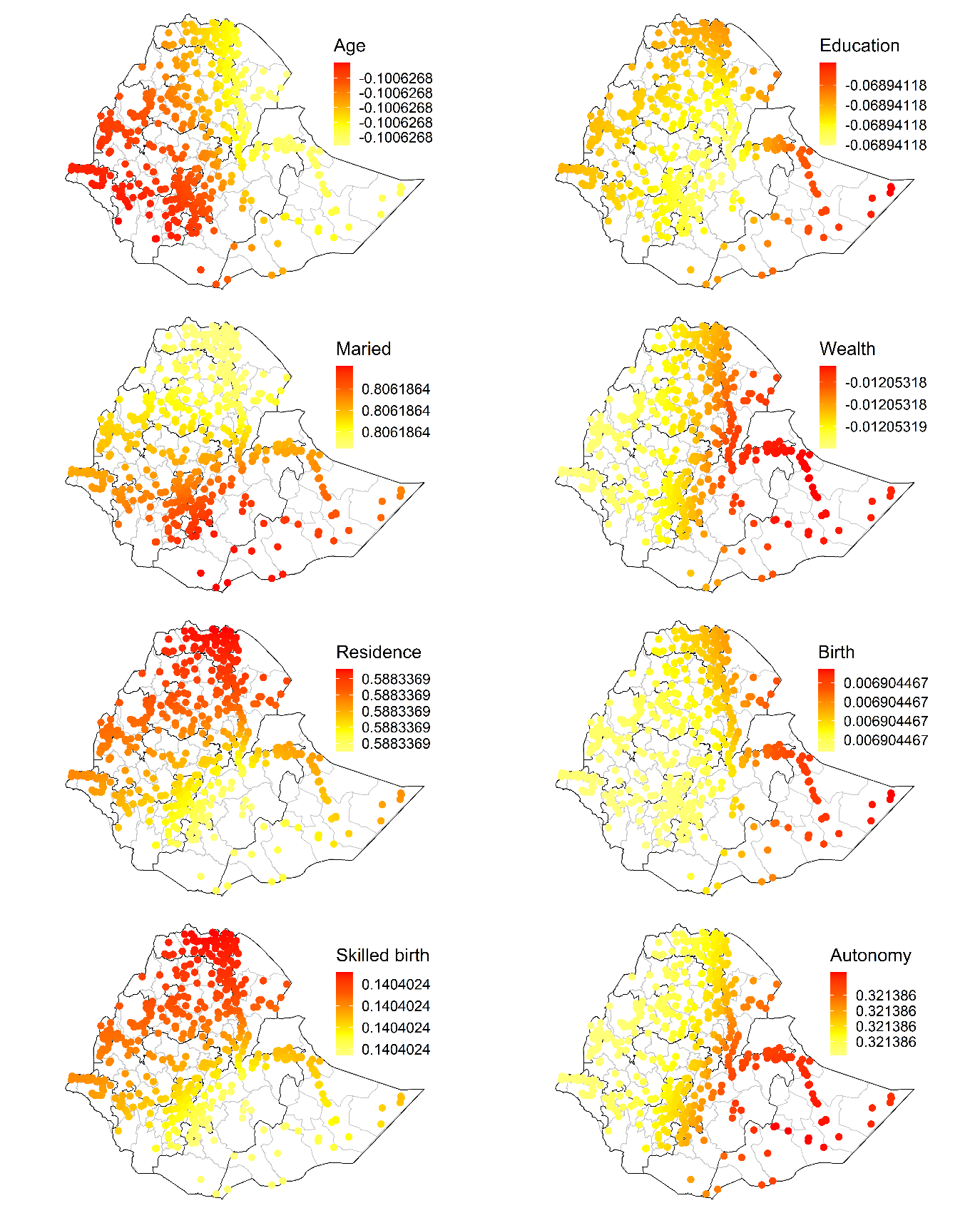
**

**Figure S2:** Geographically Weighted Poisson Regression result of variables that have no statistically significant geographically varying relationship with perinatal mortality in Ethiopia, using Demographic and Health Survey 2016 data *(each dot indicates a neighborhood, and the colors represent the regression coefficient locally, i.e., the magnitude and direction of association with the perinatal mortality at that geographic location.)*

**
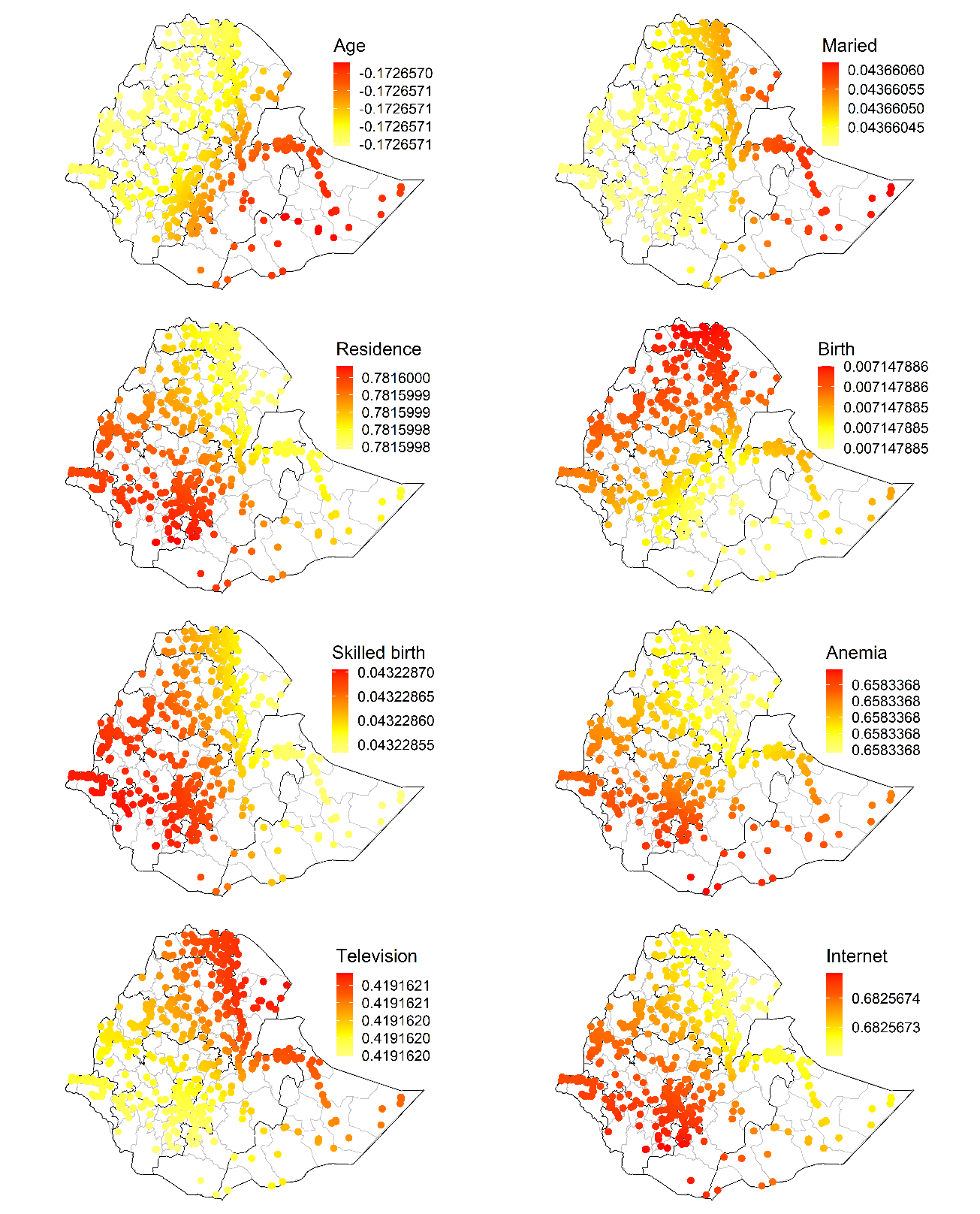
**

**Figure S3:** Geographically Weighted Poisson Regression result of variables that have no statistically significant geographically varying relationship with pregnancy-related mortality in Ethiopia, using Demographic and Health Survey 2016 data *(each dot indicates a neighborhood, and the colors represent the regression coefficient locally, i.e., the magnitude and direction of association with the pregnancy-related mortality at that geographic location.)*


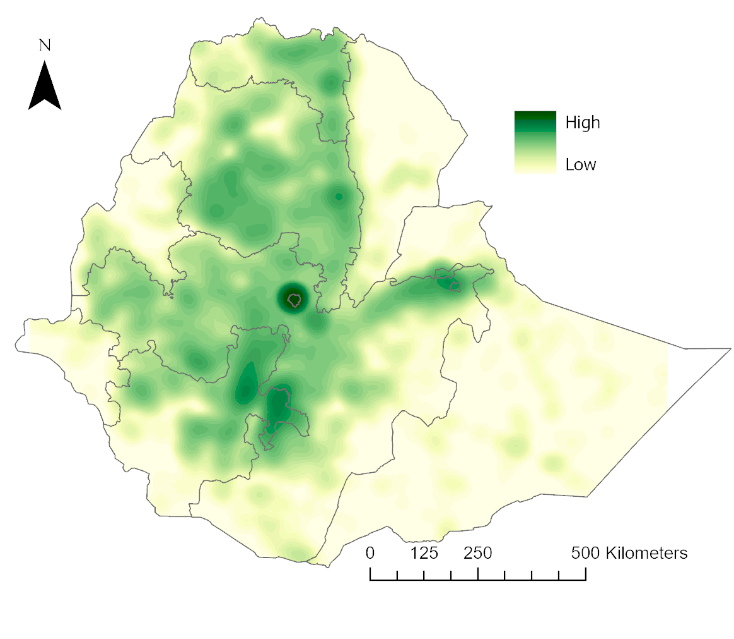


**Figure S4:** Health facility density score Kernel Density Estimation of health facilities in Ethiopia, 2016.


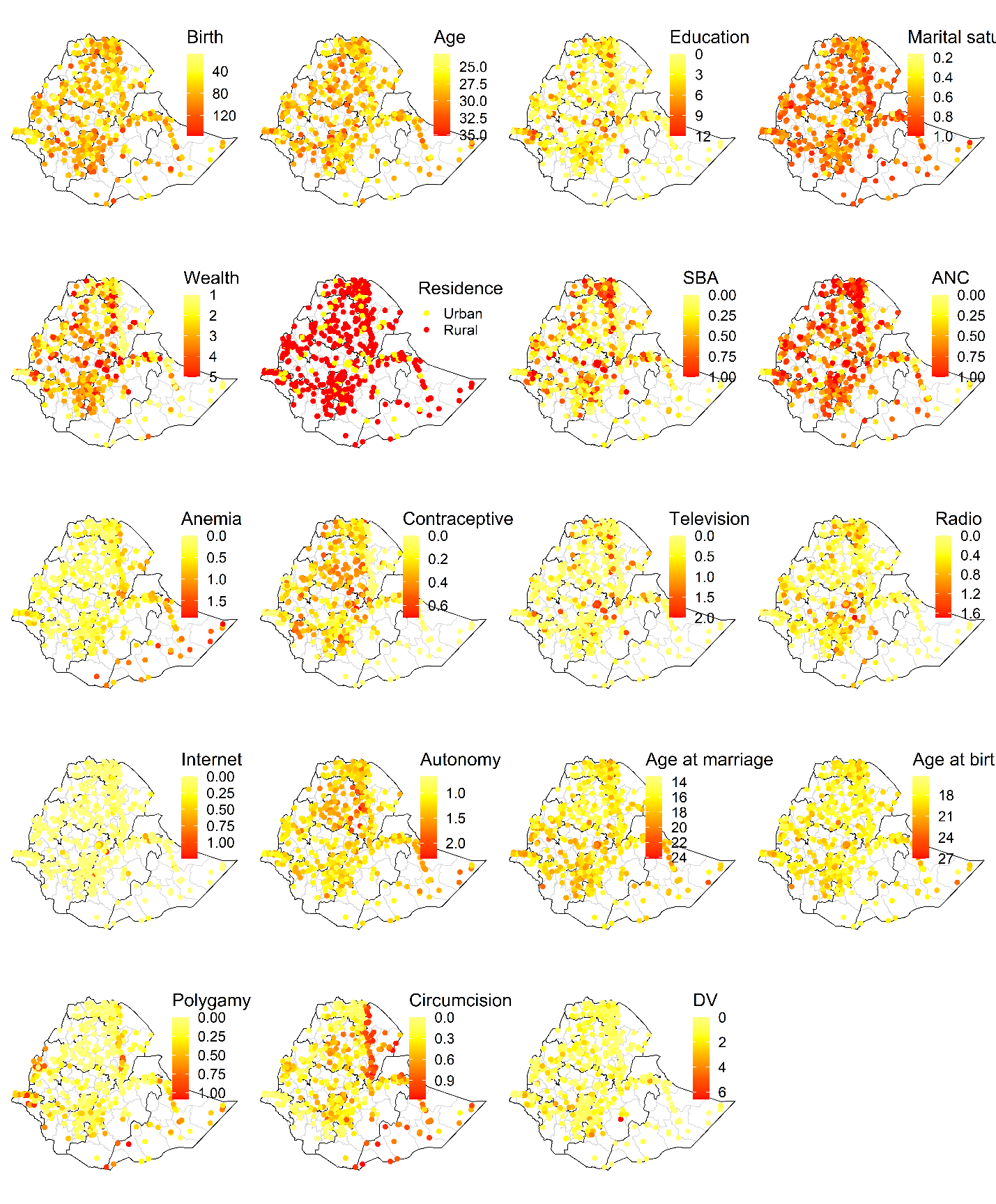


**Figure S4:** Distribution of explanatory variables across different areas in Ethiopia; 2016. *(Age = mean age of women in the neighborhood; Education = mean year of education in the neighborhood; Marital status = Proportion of women married/living together in the neighborhood; Listening radio = mean listening radio score in the neighborhood; Watching television = mean watching television score in the neighborhood; Internet = mean internet usage score in the neighborhood; Birth = mean number of number of childbirths a woman has in the neighborhood; Contraceptive = Proportion of women currently using any contraceptive method in the neighborhood; Age at marriage = mean age at first marriage of women in the neighborhood; Age at first birth = mean age at first birth of women in the neighborhood; Anemia = mean anemia score in the neighborhood; Polygamy = mean number of other wives husbands have in the neighborhood; Circumcision = mean circumcision score in the neighborhood; Autonomy = mean autonomy score in the neighborhood; DV = mean domestic violence score in the neighborhood; ANC = Proportion of women who received antenatal care during their last pregnancy in the neighborhood; SBA = Proportion of women who had a skilled birth attendance during her last delivery in the neighborhood; Wealth = mean wealth index in the neighborhood.)*
